# Biological and physiological characteristics of human cumulus cells in adherent culture condition

**DOI:** 10.18502/ijrm.v18i1.6189

**Published:** 2020-01-27

**Authors:** Somayyeh Sadat Tahajjodi, Ehsan Farashahi Yazd, Azam Agha-Rahimi, Reza Aflatoonian, Mohammad Ali Khalili, Mahnaz Mohammadi, Behrouz Aflatoonian

**Affiliations:** ^1^Stem Cell Biology Research Center, Yazd Reproductive Sciences Institute, Shahid Sadoughi University of Medical Sciences, Yazd, Iran.; ^2^Research and Clinical Center for Infertility, Yazd Reproductive Sciences Institute, Shahid Sadoughi University of Medical Sciences, Yazd, Iran.; ^3^Department of Reproductive Biology, School of Medicine, Shahid Sadoughi University of Medical Sciences, Yazd, Iran.; ^4^Department of Endocrinology and Female Infertility, Reproductive Biomedicine Research Centre, Royan Institute for Reproductive Biomedicine, ACECR, Tehran, Iran.; ^5^Department of Advanced Medical Sciences and Technologies, School of Paramedicine, Shahid Sadoughi University of Medical Sciences, Yazd, Iran.

**Keywords:** Cumulus cells, Conditioned medium, In vitro maturation, In vitro gametogenesis, Niche.

## Abstract

**Background:**

Cumulus cells, as oocyte nurse cells, provide a suitable microenvironment with growth factors and cellular interactions required for oocyte maturation. Thus, these cells may serve as a natural niche for *in vitro* studies of female germ cell development. Cumulus cells may help attain a better understanding of the causes of infertility in women and eventually improve the outcomes of cases that respond poorly to standard infertility treatment.

**Objective:**

The aim of this study was to isolate, culture, and investigate the biological characteristics of human cumulus cells.

**Materials and Methods:**

In this experimental study, cumulus cells were isolated, cultured, and characterized using reverse transcription-polymerase chain reaction analyses of specific genes including *FOXL2*, *CYP19A1*, *FSHR*, *AMHR*, and *LHR. *The presence of vimentin, a structural protein, was examined via immunofluorescent staining. Moreover, levels of anti-mullerian hormone (AMH) and progesterone secretion by cumulus cells were measured with ELISA after 2, 4, 12, 24, and 48 hr of culture.

**Results:**

In adherent culture, human cumulus cells expressed specific genes and markers as well as secreted AMH and progesterone into the medium.

**Conclusion:**

Cumulus cells secrete AMH and progesterone in an adherent culture and might be applicable for *in vitro* maturation (IVM) and *in vitro* gametogenesis (IVG) studies.

## 1. Introduction

Cumulus cells serve as oocyte nurse cells in the cumulus-oocyte complexes (COCs) during oogenesis *in vivo.* They provide a suitable microenvironment with growth factors and cellular interactions crucial for oogenesis (1). Moreover, specialized gap junctions between the human oocyte and cumulus cells allow the bidirectional signal exchange and transport of metabolic molecules, which are essential for the production of competent oocytes that can complete meiosis as well as support fertilization and embryo development (2-4). Owing to their features and availability from oocyte pick-up, cumulus cells are used in co-culture systems to improve the outcomes of *in vitro* maturation (IVM), oocyte fertilization, and embryo development. IVM is recommended for patients with polycystic ovary syndrome (PCOs) and proposed as a means of preserving the fertility of young patients at a high risk of future infertility due to aggressive chemotherapy (5, 6).

The efficiency of current IVM techniques in terms of the quality of mature oocytes, the developmental potential of embryos, and the success rates of pregnancy and live births is less than that of *in vitro *fertilization (IVF) cycles using full hormonal protocols (7). In a study in Buffalo, co-culture with cumulus cells could restore the developmental potential of denuded oocytes and compensate for the deficiency of conventional IVM methods (8). Other studies have reported undesirable effects on IVM following the removal of cumulus cells in murine (9), bovine (10), and porcine (11) models. In pigs, denuded oocytes co-cultured with cumulus cells showed higher rates of first polar body formation and survival than routine IVM (12). In humans, co-culture with cumulus cells could promote oocyte maturation (13, 14) and improve the embryo implantation and pregnancy rates in IVF cycles through secretion of various growth factors and embryotrophic factors, such as nutrients, substrates, and cytokines, as well as the detoxification of the culture medium through the removal of harmful substances, such as heavy metals, ammonium, and free radicals (15-17).

In addition to co-culture, the use of cumulus cells conditioned medium, which contains cytokines, growth factors, amino acids, and antioxidants, substantially improves IVM outcomes (14, 18). Since defective oogenesis leads to infertility in some couples, *in vitro* generation of female germ cells could be an option for future female infertility treatment. Some studies have reported improved *in vitro* germ cells derived from co-culture with cumulus cells and from culture in granulosa and cumulus cell conditioned medium (1, 19, 20).

In this study, human cumulus cells were isolated and cultured and their biological features were identified using reverse transcription polymerase chain reaction (RT-PCR) analyses of specific genes including *FOXL2*, *CYP19A1*, *FSHR*, *AMHR*, and *LHR*. Moreover, the presence of vimentin, a structural protein, was examined by immunofluorescent (IF) staining. In addition, the levels of anti-mullerian hormone (AMH) and progesterone secretion by cumulus cells into the cultured medium were measured after 2, 4, 12, 24, and 48 hr of culture using ELISA.

## 2. Materials and Methods

### Study design and materials

This was an experimental study. Chemicals and reagents were purchased from Sigma Aldrich Co. (UK). Culture media and supplements were purchased from Invitrogen Co. (UK) unless otherwise stated.

### Isolation and primary culture of human cumulus cells

Cumulus cells were obtained from infertile couples that had enrolled in an assisted reproductive technology (ART) cycle at the Research and Clinical Center for Infertility, Yazd Reproductive Sciences Institute. The infertile couples with male infertility etiology were selected for human cumulus cells isolation and culture. Following oocyte retrieval, the cumulus mass was carefully separated from COCs using hypodermic needles and treated with 300 μ l/ml hyaluronidase for 30s. For enzyme inactivation, Dulbecco's Modified Eagle Medium (DMEM) was added, and cells were washed using centrifugation at 200 × *g* for 10 min. The pellet was separated into 25-cm ${}^{2}$ tissue culture flasks containing DMEM supplemented with 20% fetal bovine serum (FBS) and 1 μ l/ml pen-strep and incubated at 37°C and 6% CO ${}_{2}$ . Conditioned medium was collected every 3 days from the flasks that achieved 80% confluence, filtered, and stored at - 20°C.

### RNA extraction, cDNA synthesis, and RT-PCR

Trypsin-EDTA (1 ml) was added to the culture and incubated for 3 min at 37°C to detach the cumulus cells. Cumulus cells were collected at 300 × *g* for 5 min in a microfuge tube. The pellet was re-suspended in 1 ml TRI reagent; tubes were vortexed to completely lyse the cells. RNA extraction was performed according to the manufacturers' recommended protocol. RNA quantity was determined using a Nano Drop spectrophotometer (Thermo Scientific Co., UK). A DNase treatment kit was used to remove genomic DNA contamination from the RNA sample. Total RNA was used for cDNA synthesis using the first strand cDNA synthesis kit (Takara Co., Japan). Total reaction volume in each RT-PCR cycle was 20 μ l [1 μ l of cDNA, 1 μ l primers, 6 μ l RNase and DNase free water, and 12 μ l mastermix (AMPLIQON Co., Denmark)]. Primer sequences used are shown in Table I. Agarose gel (2%) stained with ethidium bromide was used for the detection of PCR products.

### IF staining

IF staining was used to evaluate vimentin expression (ab5733; MILLIPORE Co., USA) in cultured cumulus cells, as described elsewhere (21).

### Detection of hormone secretion

Medium from cumulus cells primary culture was collected at 2, 4, 12, 24, and 48 hr after culture. AMH and progesterone levels were measured using ELISA (AMH: AnshLabs Co., Germany; Progesterone: monobind Co., USA).

**Table 1:** List of primers used for RT-PCR


**Gene name**	**Forward primer (5 ' -3 ' )**	**Reverse primer (5 ' -3 ' )**	**Product size (bp)**
*AMHR *(NM_001164691.2)	CTTGACCCAGTACACCAGTG	ACACGATCCATCTTCCCGAA	172
*FOXL2 *(NM_023067.4)	TTATGTCCTCCTGTGCTCAC	AAAGAGACGAGCCCAGTAGA	98
*CYP19A1*(NM_001347256.1)	ACTACAACCGGGTATATGGAGAA	TCGAGAGCTGTAATGATTGTGC	119
*FSHR *(NM_181446.2)	TCTGGCAGAAGACAATGAGTCC	TGAGGATGTTGTACCCCATGATA	157
*LHR *(NM_000233.4)	TCCCTGTCAAAGTGATCCCA	CCGGGCTCAATGTATCTCAG	175
* β 2M *(NM_004048.2)	AGATGAGTATGCCTGCCGTG	TGCGGCATCTTCAAACCTC	106
* Annealing temperature of 60°C was used for all primers			

### Ethical consideration

Cumulus cells were obtained following informed consent from male factor infertile couples that had enrolled into an ART cycle at the Research and Clinical Center for Infertility, Yazd Reproductive Sciences Institute (Ethics Committee reference number: IR.SSU.MEDICENE.REC.1395.94).

## 3. Results

### Isolation and primary culture of human cumulus cells

**Figure 1:**
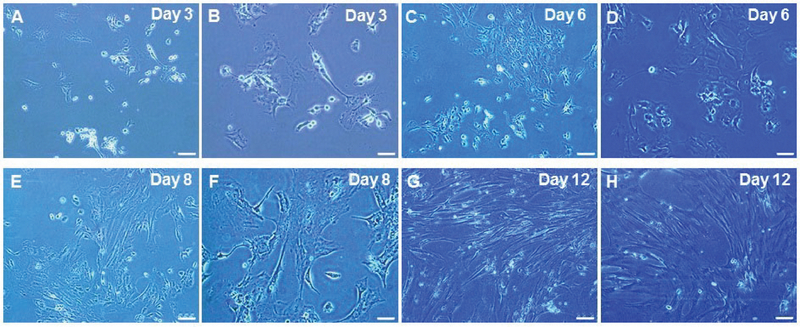
Primary culture of cumulus cells at days 3 (A, B); 6 (C, D); 8 (E, F); and 12 (G, H) at different magnifications. Scale bar: 100 μ m (A, C, E, and G), 50 μ m (B, D, F, and H).

Cumulus cells from COCs following mechanical and enzymatic treatments were cultured in 25-cm ${}^{2}$ tissue culture flasks containing DMEM supplemented with 20% FBS and 1 μ l/ml pen-strep and incubated at 37°C and 6% CO ${}_{2}$ . Cumulus cells started to proliferate over subsequent days (Figure 1A and 1B). The proliferating cells spread out from the attached cumulus clusters (Figure 1C-1F). Cumulus cells clusters spread over the flask 12 days after primary culture (Figure 1G and 1H).

### Characterization of human cumulus cells

**Figure 2:**
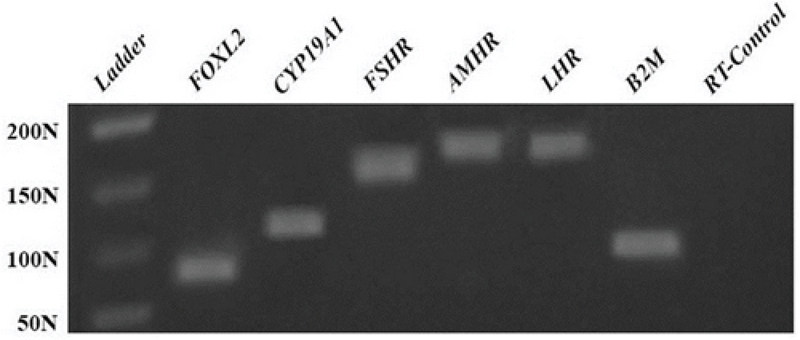
Gene expression profile for cultured human cumulus cells. * N= Nucleotide.

**Figure 3:**
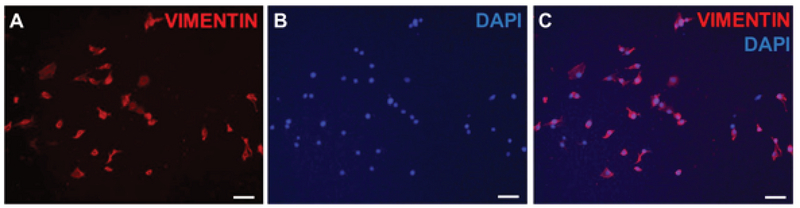
IF staining of human cumulus cells for vimentin (A), DAPI (B), and vimentin plus DAPI (C). Scale bar: 100 μ m.

Cumulus cells showed positive expression for specific genes (Figure 2). The expression of *FOXL2*, *CYP19A1*, *FSHR*, *AMHR*, and *LHR* was examined by RT-PCR, and all genes were expressed in cumulus cells isolated from COCs and cultured in 25-cm ${}^{2}$ tissue culture flasks containing DMEM. Vimentin, a type III intermediate filament, is a structural protein; cumulus cells showed positive expression of this cytoskeletal marker in IF staining (Figure 3).

### Detection of hormone secretion

**Figure 4:**
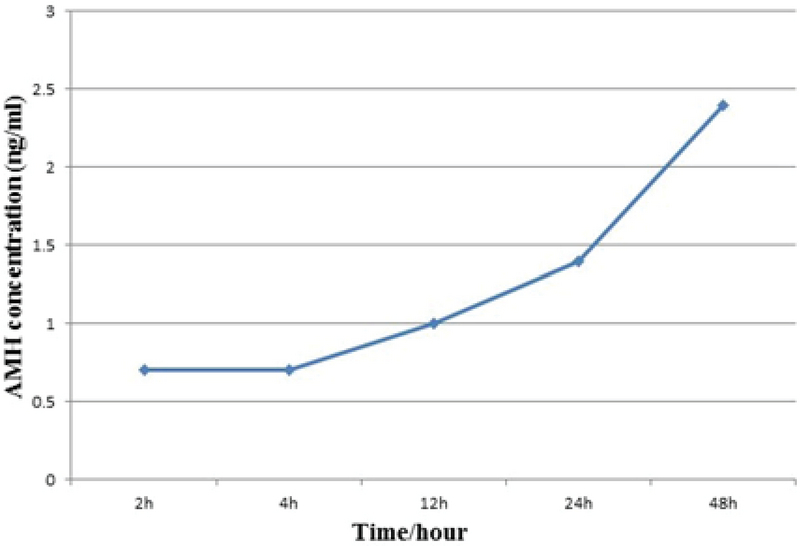
AMH concentration (ng/ml) following 2, 4, 12, 24, and 48 hr of culture.

**Figure 5:**
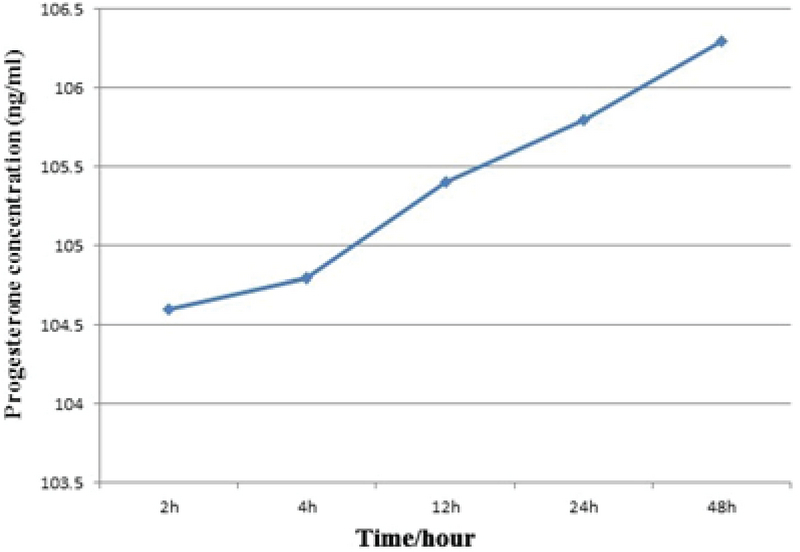
Progesterone concentration (ng/ml) following 2, 4, 12, 24, and 48 hr of culture.

AMH and progesterone levels were measured using ELISA 2, 4, 12, 24, and 48 hr after cumulus cell culture. Up to the 4-hr measurement, the AMH level did not change but it increased gradually after 4 hr (Figure 4). Progesterone levels increased steadily during the experimental period (Figure 5).

## 4. Discussion

In this study, we isolated and cultured human cumulus cells. The cells were then characterized by assessing the gene expression profiles of *FOXL2*, *CYP19A1*, *FSHR*, *AMHR*, and *LHR* using RT-PCR. Furthermore, IF staining was used to identify the vimentin-expressing cumulus cells in the culture. ELISA indicated the cumulus cells secreted AMH and progesterone. AMH and progesterone levels in the culture medium gradually increased after 2, 4, 12, 24, and 48 hr of culture.

Previous studies have reported that co-culture with human cumulus cells or culture in cumulus cells conditioned medium can improve IVM outcomes and thus the embryo implantation and pregnancy rates in IVF programs (7, 8, 12-18). Therefore, the biological characteristics of human cumulus cells following culture were investigated in this study.

Culture of cumulus cells of humans (2, 13, 14) and other mammals (1, 8, 12) has been previously reported. Monolayers or clumps of cumulus cells were used for co-culture, and clumps of cultured cumulus cells were used for obtaining conditioned medium (14, 18). In this study, cumulus cells were separated from COCs using mechanical and enzymatic treatments, and individual cells were seeded into tissue culture flasks. Furthermore, different culture media, such as TCM-199 (8, 12, 13), BM1 medium (14), and animal-free serum-free defined medium (SPE-IV/EBM-500; ABCell-Bio) (2), have been used in previous studies. In a study in Buffalo, cumulus cells were cultured in DMEM supplemented with 10% FBS to harvest conditioned medium for the differentiation of embryonic stem cells (ESCs) into germ cells (1). Herein, we used similar culture conditions for the primary culture of human cumulus cells.

Many previous studies have demonstrated that the functions and products of five genes (*FOXL2*, *CYP19A1*, *FSHR*, *AMHR*, and *LHR)* are detected in the cumulus cells. For instance, FOXL2 (Forkhead Box L2) supports folliculogenesis following puberty by regulating cumulus cell differentiation (22). This gene is a transcription factor, and its expression is important for fetal ovarian development and commitment. FOXL2 suppresses the expression of SOX9, which prevents testes formation (23). *CYP19A1* expression in cumulus cells produces the enzyme responsible for estrogen biosynthesis, folliculogenesis regulation, follicular development, and ovarian function (24). The down-regulation of *AMH* expression and up-regulation of aromatase *CYP19A1* expression influence the capacity of the follicle to produce estradiol (25). FSH is an important hormone in female reproduction, and it is necessary for maturation and gamete production at puberty and during the reproductive phase of life. The FSH receptor (*FSHR*) is expressed in a similar pattern in both cumulus and granulosa cells. In addition, cumulus cells demonstrate measurable expression of *LHR *(26, 27). Catteau-Jonard and colleagues demonstrated that expression of the AMH receptor (*AMHR*) is a marker for AMH bioactivity in cumulus cells. The mRNA expression levels of *LHR*, *FSHR*, *AMHR*, and *CYP19A1* are very closely associated with one another (26, 28).

Therefore, due to the function of the abovementioned gene products, their expression was assessed following the isolation and culture of the human cumulus cells in a monolayer culture condition. Some studies used cultured cumulus cells as a feeder layer for the differentiation of pluripotent stem cells (19, 20) and induced pluripotent stem cells (iPSCs) as well as for comparison with human foreskin fibroblasts (HFFs) (2). Similar to our study (Figure 3), human cumulus cells express vimentin, as detected by IF staining.

The secretion of AMH and progesterone was measured by ELISA. P450scc and 3 β HSD are two enzymes that convert cholesterol to progesterone, and cumulus cells express these genes during ovulation. Furthermore, these cells possess a cholesterol synthesis pathway and produce progesterone from the *de novo* synthesized cholesterol (29, 30). The level of progesterone secreted by porcine COCs into the culture medium was increased when the number of porcine COCs increased in well-plate cultures (31).

AMH, a member of the transforming growth factor-beta (TGF β ) superfamily, is an important regulator of mammalian follicular development. The secretion of AMH by granulosa cells gradually increases during the development of the primary follicles to large preantral and small antral follicles and decreases thereafter (32, 33). The secretion of AMH by granulosa cells declines as the follicle grows; however, cumulus cells have a relatively high capacity to synthesize AMH during the final stages of folliculogenesis (34).

Our data confirm the previous findings regarding the secretion levels of progesterone and AMH (Figures 4 and 5). Adding progesterone and AMH to the culture medium may improve the quality of IVM and subsequent embryo development to morula and blastocyst (35).

## 5. Conclusion

First, human cumulus cells were isolated and cultured in a monolayer adherent culture condition. Subsequently, the biological and physiological characteristics of the cells were identified by gene expression profile assessment and hormone secretion detection. Human cumulus cell cultures and their conditioned medium are suitable materials for further *in vitro* studies to mimic human oogenesis processes in IVM and in *in vitro* gametogenesis (IVG) applications.

##  Conflict of Interest

The authors declare no conflicts of interest.
